# Assessment of Shape Variation Patterns in *Triatoma infestans* (Klug 1834) (Hemiptera: Reduviidae: Triatominae): A First Report in Populations from Bolivia

**DOI:** 10.3390/insects11050274

**Published:** 2020-04-30

**Authors:** Carolina Vilaseca, Marco A. Méndez, Carlos F. Pinto, Hugo A. Benítez

**Affiliations:** 1 Laboratorio de Ecología Química, Universidad Mayor Real y Pontificia San Francisco Xavier de Chuquisaca, Sucre, Bolivia; vilaseca_c@yahoo.com.ar (C.V.); pinto.carlos@usfx.bo (C.F.P.); 2 Laboratorio de Genética y Evolución, Facultad de Ciencias, Universidad de Chile, Santiago 6640022, Chile; mmendez@uchile.cl; 3 Laboratorio de Ecología y Morfometría Evolutiva, Centro de Investigación de Estudios Avanzados del Maule, Universidad Católica del Maule, Talca 3466706, Chile

**Keywords:** *T. infestans*, inter-Andean, Chaco, wing shape, morphometrics

## Abstract

The morphological variations of four populations of geographically isolated *Triatoma infestans* located in the area of inter-Andean valleys and Chaco of Chuquisaca, Bolivia, were evaluated. Fifty-three females and sixty-one males were collected in the peri-domicile and analyzed with geometric morphometrics tools to study the patterns of the head and wing shape variation. The principal component analysis and canonical variate analysis revealed morphological variations between the populations studied, which were then confirmed by the permutation test of the differences between populations using Mahalanobis and Procustes distances. The multivariate regression analysis shows that the centroid size influences the shape of the heads and wings. *T. infestans* of the inter-Andean valleys are longer in the head and wings compared to the population of the Chaco. We propose that the geometric shape variation may be explained by geographical changes in climatic conditions, peri-domiciliar habitats, food source quality, and the use of insecticides.

## 1. Introduction

*Triatoma infestans* (Klug 1834) (Hemiptera: Reduviidae: Triatominae) is a hematophagous insect vector responsible for the transmission of Chagas disease in South America [1]. This insect is found in rural areas; it inhabits human housing and domestic animals [2]. In Bolivia, wild foci have been identified in the Andean and Chaco areas [3,4,5].

Chagas disease is an endemic parasitosis that affects between 6 and 7 million people in the world, most of them in Latin America. The disease leads in some cases to very debilitating injuries in the heart and intestinal tract [6]. The causative agent is a flagellated protozoan called *Trypanosoma cruzi*, the transmission is by Triatomine insects. In Bolivia, the transmission by insect vector represents 80% of cases of the disease and the remaining 20% correspond to a trans-placental route and blood transfusions [4]. 

Chagas disease in Bolivia covers large geographic areas, from the Andean valleys to the Chaco region, of which 60% is considered as an endemic area. Only the highlands would be a barrier to the spread of the disease by the main insect vector *Triatoma infestans* which has adapted to domiciliar and peri-domiciliar environments [3]. In Bolivia, the average percentage of infected people in endemic areas is 20%; Chuquisaca has the highest average at 29.5% [7]. Epidemiological studies of the disease have been carried out in the region, but there are few investigations related to the insect vector. Until now, the existence of sylvatic foci of *T. infestans* has been unknown in Chuquisaca, although the Chaco area is currently under analysis [3,8,9]. 

In Chuquisaca, *T. infestans* frequents both intra-domiciliary and peri-domiciliary habitats. The chicken coops, pigpens, goats, and rabbits located a few meters from houses serve as an important refuge and potential infestation and colonization points [3]. The constructions of the corrals and chicken coops differ regionally; in the inter-Andean valleys they are structures built with blocks of earth forming solid walls, while in the Chaco wooden sticks and palm roofs form them [3]. The intra-domiciliary infestation rate in the inter-Andean valleys is less than 3%, but in the peri-domicile it is greater than 7%, while in the Chaco both rates are higher than 14%, which could be explained by the wretched constructions leading to rapid degradation of insecticides [9,10]. 

Due to its epidemiological importance, population studies of *T. infestans* have relied on molecular tools to investigate the routes and mechanisms of dispersion in South America by means of chromosomes, isoenzymes, mitochondrial and ribosomal markers [6,11,12,13]. Molecular tools have also been used to investigate re-infestation processes by means of microsatellite markers [14,15,16]. Investigations in traditional and geometric morphometrics have been used for ecology and systematics studies [13,16], domestication studies related to sexual dimorphism [17], feeding behavior in natural habitats [18], combined morphometric studies with isoenzymes electrophoresis in re-infestation processes [19], as well as studies about *T. infestans* population structuring [20]. 

In the last decades, investigations about Triatomines using morphometrics have analyzed the morphological and genetic relationships regarding multiple host infestation [21,22]. Natero et al. [22] found that the effect of morphological shape modification in Triatomine species is frequently linked to the type and quality of blood ingested during nymph instars and adult stages. The use of multivariate tools in the characterization of Triatomine morphological variation has contributed to a decrease in identification errors, as well as improving its effects on vector biology [23,24,25,26].

The aim of this study is to use geometric morphometric (GM) analysis with landmark-based methods of 2D cartesian coordinates in order to identify changes in morphology in isolated populations from the area of Chuquisaca in Bolivia.

## 2. Materials and Methods

### 2.1. Study Area

Chuquisaca is located in Southern Bolivia; it has two geographical areas: the sub-Andean sector and the Chaco plain. The former is enclosed by two mountain ranges: the eastern and central mountain ranges; high and low inter-Andean valleys are found in the mountainous relief. In these areas, the mountains get in the way of the humid air coming from the east, causing abundant rain. Low inter-Andean valleys have high, medium, and low mountains with small hills, and a humid environment. The high inter-Andean valleys are over 2900 m above sea level, with an annual average temperature around 18 °C with high plateau characteristics. The wet Chaco is a landscape of steep mountains; the highest ones do not exceed 2600 m above sea level, and at the base of the mountains, around 900 m above sea level, there is a warm and humid climate; this whole area belongs to the Tucumano Boliviano forest. The dry Chaco areas are to the east of the Cordillera Oriental, which is a region of flat arid lands [7]. The Bolivian Chaco next to the border with Paraguay and Northern Argentina, characterized by a warm climate with temperatures above 30 °C during summer, is the Bolivian Boreal Chaco [7].

**Figure 1:**
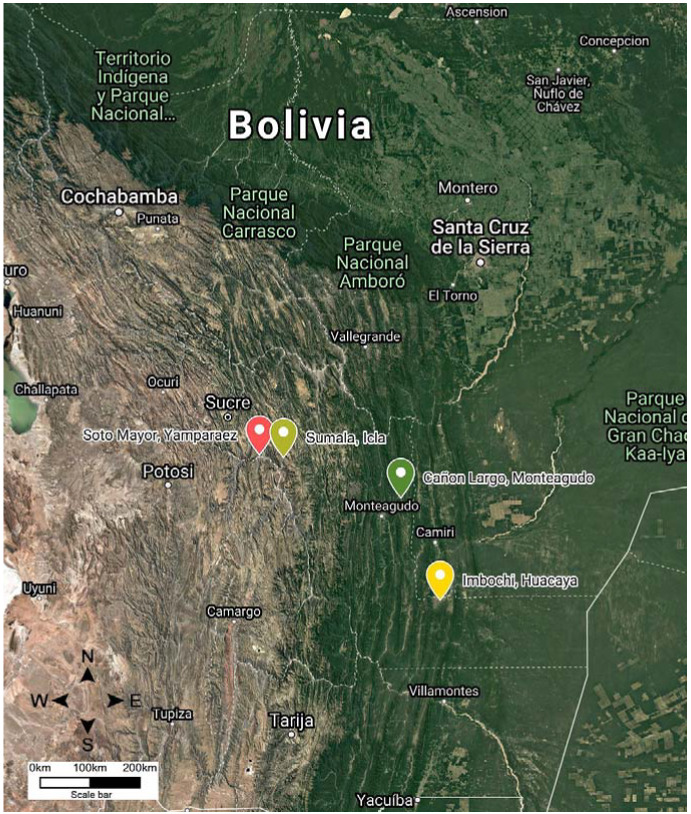
Coordinate information from the Military Institute of Geography for the four locations where *Triatoma infestans* populations were sampled in Chuquisaca: Cañón Largo/Monteagudo (green), Imbochi/Huacaya (yellow), Sotomayor/Yamparáez (red), Sumala/Icla (light green).

In this study, *T. infestans* were collected in four geographical locations in the Chuquisaca region of Bolivia: Yamparáez/Sotomayor (inter-Andean valley), Icla/Sumala (inter-Andean valley), Monteagudo/Cañón Largo (wet Chaco), and Huacaya/Imbochi (dry Chaco) (Figure 1). The regions varied in terms of both temperature and relative humidity, where the inter-Andean valley has lower temperatures and higher relative humidity, and wet Chaco has higher temperatures and lower relative humidity. Table 1 summarizes the climatic characteristics of the four geographical locations where *T. infestans* specimens were collected. The characteristics of the geographical zones were obtained from the Military Institute of Geography of Bolivia and they are described in Table 1.

### 2.2. Sampling

A total of 53 females and 61 males were collected from peri-domiciliary (chicken coops and domestic animal pens). The collection was made between May and September 2017. The capture of Triatomines was carried out by active search in chicken coops and sheep and pigpens for one hour, and adult insects were stored in a plastic container. At least 11 insects of the same sex were collected per location. They were preserved in the laboratory, in alcohol (96%). Then the wings were slide-mounted using Euparal^®^. All wings and heads were photographed using a Celestron Handheld Digital Microscope pro 5MP.

### 2.3. Morphometric Analyses

**Figure 2:**
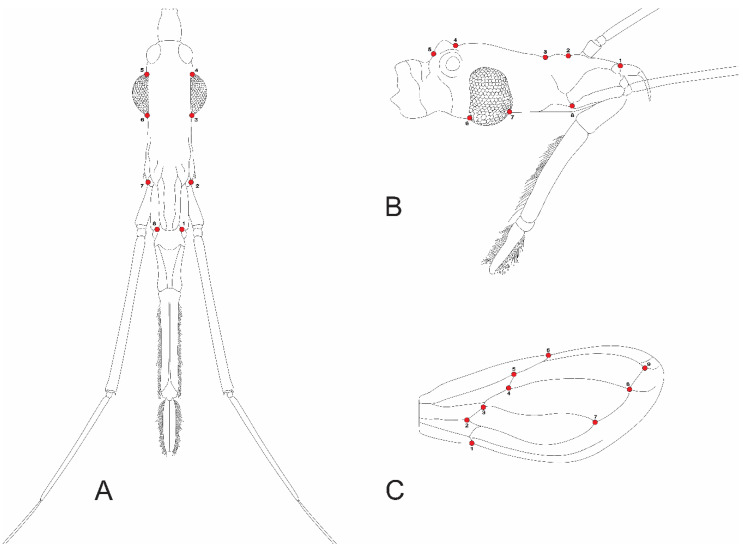
Indication of landmarks in two head view and wing of *Triatoma infestans*. (**A**) Dorsal view; (**B**) lateral view; (**C**) wing view.

Eight landmarks were selected for both the dorsal and lateral views of the head (Figure 2A,B). Nine landmarks were selected for both the right and left wings (Figure 2C).

The software program tpsDIG V232 was used to digitize landmark coordinates, as well as capture outlines [27]. All morphometric measurements were conducted using MorphoJ software V. 1.06d [28]. For all landmarks, the shape information was extracted using a Procrustes superposition analysis, which is a procedure that removes the information on size, position, and orientation to standardize each specimen according to centroid size [29]. 

The measurement error (ME) was calculated using a Procrustes ANOVA, in order to detect digitizing errors in morphometric data. For this procedure, the original dataset was compared with a control of repeated measures, and the values of the means squares (MS) of the individual values were compared with the error (dataset of the repeated measurement) [30,31].

To characterize the variation related with shape dimensions, a principal component analysis (PCA) was carried out based on the covariance matrix of shape of the datasets of dorsal view, lateral view and wing view. Canonical variate analysis (CVA) methods were used as a method to amplify the shape variation and stand out the differences between *T. infestans* localities [32,33]. Different morphological distances were calculated and reported (Mahalanobis and Procrustes distances) with their respective *p*-values after a permutation test (10,000 runs). A multivariate regression of size (independent variable) on shape (dependent variable) was performed to analyze if the size has an influence on the shape distribution of *T. infestans* populations. All the analyses were performed using the software MorphoJ 1.06d [28].

## 3. Results

The Procrustes ANOVA for assessing the measurement error of both views of head, dorsal and lateral, showed that the mean square for individual variation exceeded the measurement error: dorsal MS error: 0.000114 < MS individual: 0.000159, lateral MS error: 0.000206 < MS individual: 0.000245. The measurement error in the wings showed that the mean square for individual variation exceeded the measurement error (MS error: 0.0000949 < MS indxside: 0.000181).

**Figure 3:**
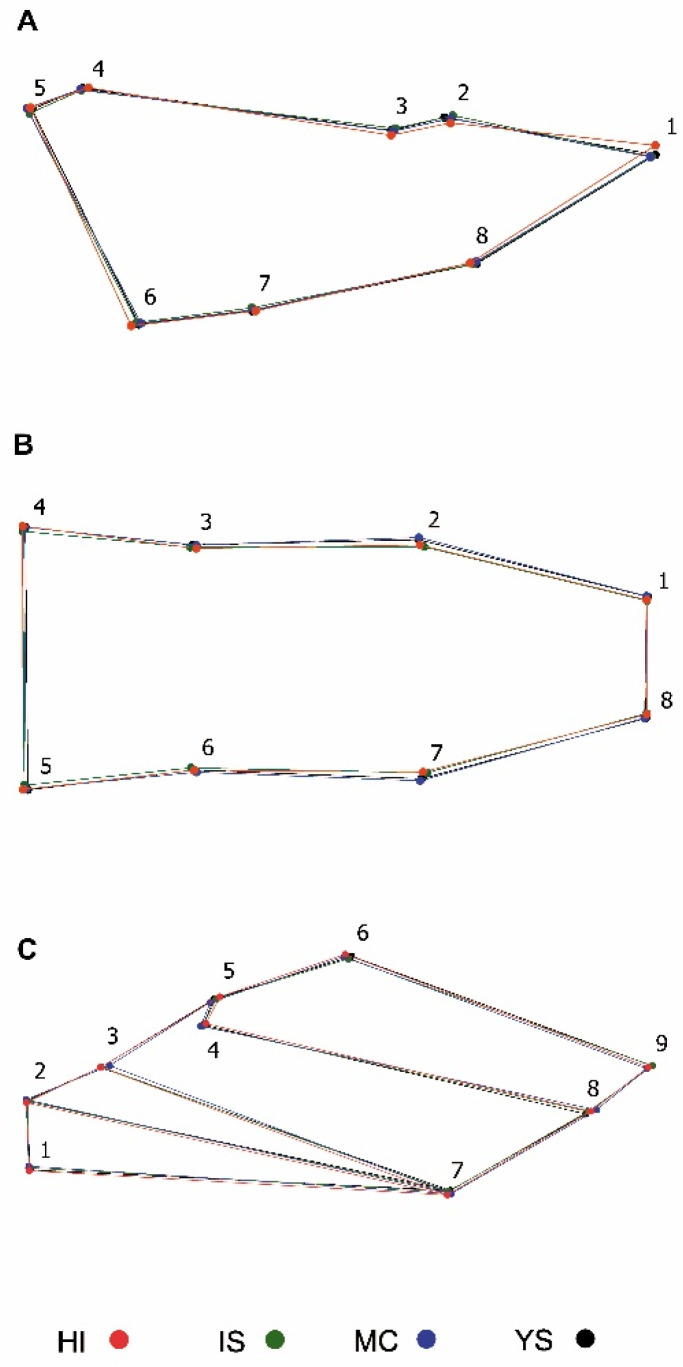
Average shape representation of the four locations of *Triatoma infestans*. (**A**) head lateral view; (**B**) head dorsal view; (**C**) wings. Red: HI (Huacaya/Imbochi); green: IS (Icla/Sumala); blue: MC (Monteagudo/Cañón), and black: YS (Yamparáez/Sotomayor).

PCA of the head showed that the three PCs accounted for 58.83% (PC1: 29.32%, PC2: 16.36%, PC3: 13.14%) of the shape variation of the dorsal and 65.40% (PC1: 36.42%, PC2: 14.76%, PC3: 14.22%) of the lateral. The PCA of the wings showed that the first three PCs accounted for 64.00% (PC1: 34.38%, PC2: 17.14%, PC3: 12.48%). In order to localize the shape variation, the average shape was extracted for the different localities, where the dorsal head view showed that populations of *T. infestans* from Yamparáez/Sotomayor differ principally on the location of landmarks 1 and 8 (apex of gena) and in landmarks 2 and 7 antenna insertion points. A narrowing in the head of males is observed compared to females. In the head lateral view, a narrowness is observed in landmarks 2 and 8 in the males and the wings particularly are differentiated by the slight widening observed in landmarks 5 and 6; 8 and 9 (Figure 3).

**Figure 4:**
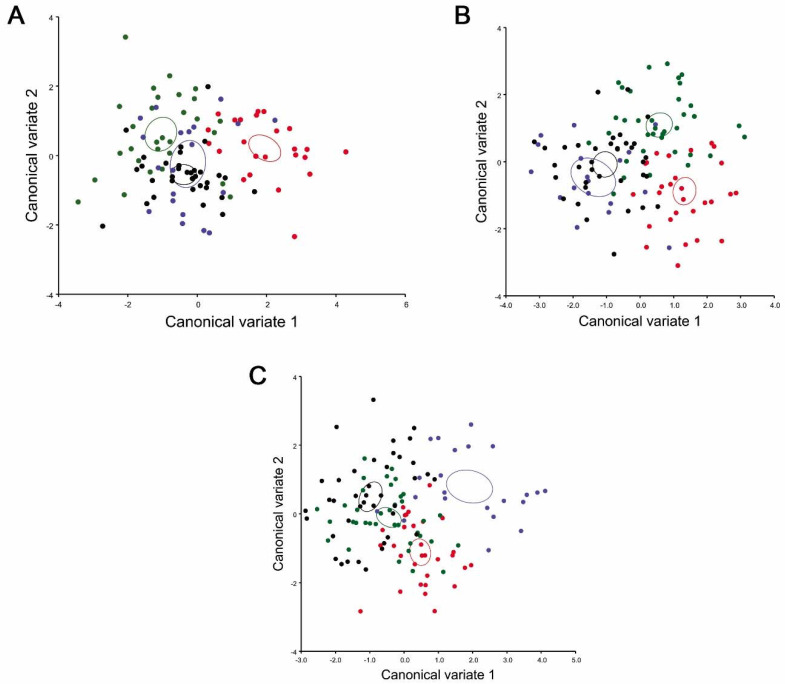
Scatterplot of the canonical variate analysis of four locations’ populations of *Triatoma infestans*. (**A**) Canonical Variate Analysis (CVA) of head dorsal view; (**B**) CVA of head lateral view; (**C**) CVA of wings. Red: HI (Huacaya/Imbochi), green: IS (Icla/Sumala) blue: MC (Monteagudo/Cañón), and black: YS (Yamparáez/Sotomayor). The confidence ellipses were computed using the multivariate mean of each population.

The scatterplot of the CVA shows a clear differentiation between the populations of *T. infestans*. Graphically, this variation can be observed between the population of inter-Andean valleys and the Chaco (Figure 4A–C). After extracting the morphological distances of the CVA, the *p*-values of Procrustes distance and Mahalanobis distance after permutations test (10,000 runs) were < 0.0001 among Huacaya/Imbochi and Yamparáez/Sotomayor, Icla/Sumala, Monteagudo/Cañón Largo (Table 2). The relationship between the morphological distances of *T. infestans* of Huacaya/Imbochi differs from other populations by the morphometric characteristics of the head and wings. Additionally, *T. infestans* of Yamparáez/Sotomayor is similar in the morphometric characteristics of the head to Monteagudo/Cañón Largo, and in the characteristics of the wings to Icla/Sumala.

**Figure 5:**
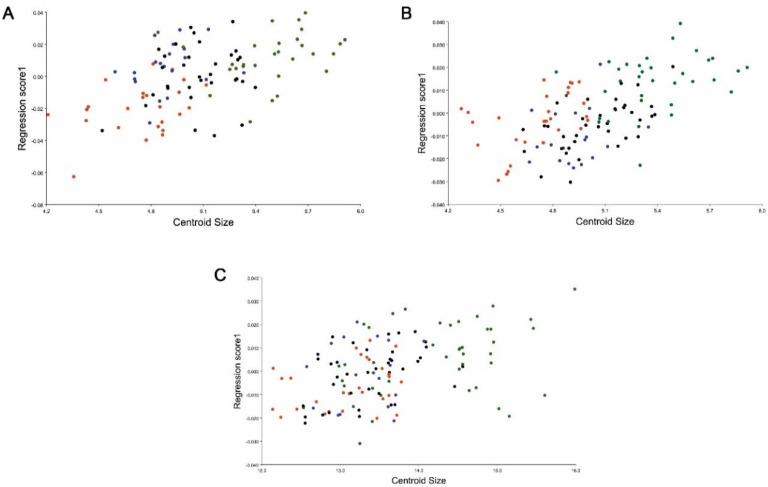
Multiple regression analysis of four locations’ populations of *Triatoma infestans,* the *y*-axis corresponds to the shape (regression scores1) and the *x*-axis to the size (centroid size). (**A**) Head, dorsal view; (**B**) head, lateral view; (**C**) wings. Orange: HI (Huacaya/Imbochi), green: IS (Icla/Sumala), blue: MC (Monteagudo/Cañón), and black: YS (Yamparáez/Sotomayor).

The multivariate regression showed that regardless of the percentage being lower, the influence of size was noticeable at the different traits evaluated where the shape variation showed a clear influence by allometry (dorsal head view 7.1622%; <0.0001, lateral head view 4.1565%; <0.0011; wings 4.3394%; <0.0001); it is possible to identify that the specimens from Icla/Sumala are bigger than the other populations in contrast with the specimens of Huacaya/Imbochi, which are the smallest (Figure 5A–C). 

## 4. Discussion

Geometric morphometrics tools were helpful in identifying shape variation associated with geographical and possibly environmental influences; this relationship in some way impacts the shape and size of *T. infestans* populations of Chuquisaca. Nevertheless, the effect of insecticides, pyrethroids, and diet may also influence patterns of asymmetry in the morphology of *T. infestans* that can vary depending on their microenvironments within the inter-Andean valleys and Chaco [21,34].

The characteristics of the constructions of pens, formed by sticks and the roofs of palm trees, make them ideal microclimates for the development of *T. infestans* in Huacaya/Imbochi, as their development is encouraged by high temperature and low relative humidity [35,36]. Monteagudo/Cañón Largo is located in the wet Chaco area; the inhabitants raise pings just as in Huacaya/Imbochi and the characteristics of the pens are also similar. However, Monteagudo/Cañón Largo has higher relative humidity, an unfavorable factor for the development of *T. infestans* and other insects in general.

Icla/Sumala and Yamparáez/Sotomayor are in the zone of inter-Andean valleys, where the mountains condense the relative humidity between 30% and 45%, which is unfavorable for the development of *T. infestans* because they can easily be infected with fungi and increase their mortality rate, and the temperature is between 18–25 °C [36]. The chicken coops and pens are made of earth blocks and thatched roofs, therefore, they are microenvironments with higher humidity that maintain low temperatures; these environmental characteristics make *T. infestans* grow larger in a process of environmental adaptation [36]. 

In our results, the centroid size (CS) significantly influences the shape of the head and wings. *T. infestans* of Icla/Sumala are larger compared to the populations of Huacaya/Imbochi; the remaining locations of the populations have an intermediate size between them. *T. infestans* of Icla/Sumala are larger and this may be due to a situation of natural selection that favors the existence of phenotypes adaptable to the adversities of the environment, and in favor of larger female body size, where fertility is a function of maternal size [37].

The larger size of the Icla/Sumala female population can also be attributed to nutritional factors because in the low inter-Andean valleys most inhabitants have peri-domestic chicken coops, and the blood of chickens makes *T. infestans* longer. Hernandez et al. [18] found that the centroid size (CS) in *T. infestans* female heads was longer compared to males in their natural habitat (peri-domestic), like chicken coops. In Huacaya/Imbochi, the dry Chaco area, the availability of a diet with human blood and pig blood permit *T. infestans* to have smaller sized heads and wings; principally, in summer the biological cycles are shortened and insects tend to have smaller sized wings [38]. The absence of walls in the pens in the Chaco zone allows *T. infestans* to easily fly to homes, leading to frequent peri-domicile to intra-domicile re-infestation, mainly at the end of summer when people usually sleep in the open air or with the door open. Studies of *T. infestans* collections with light traps revealed significant dispersal of the insect during summer [39,40].

The males of *T. infestans* populations of inter-Andean valleys have a centroid size (CS) smaller than that of females due to a residual effect of insecticides which modify the phenotype of the males, but not females, which may explain a favorable effect in intraspecific food competition for females [41]. The construction of the chicken coops and pens (blocks of earth) in the inter-Andean valleys allows for less degradation of the insecticide due to sunlight and rain [2], something that does not occur with the structures of the Chaco region (made of sticks and palm roofs). The pens of the Chaco are established as a source of re-infestation in homes; studies carried out after treatment with deltamethrin demonstrated that *T. infestans* adults disperse into homes [34]. 

*T. infestans* in the area of Yamparáez/Sotomayor presented sexual dimorphism, a thinning in the heads, and a slight widening in the wings of the males observed in comparison with the females in peri-domestic environments. According to Dujardin et al. [17], *T. infestans* and other insects, such as *Rhodnius domesticus,* present phenotypic plasticity of body size and an important mechanism to increase or decrease in size in response to short-term environmental variation, while shape variation has a genetic component [42].

These changes can be attributed to the frequent use of insecticides which leads to an adaptation process. In post-treatment studies with peri-domicile insecticides, the *T. infestans* males showed morphological variation in the head without changes in females, due to a possible intraspecific competition for food with priority to females [18,41]. Studies of sexual shape dimorphism have found that the ecological aspect of stress in populations can be reflected in reduction of the centroid size of the wings in males and the average length of the body compared to females [43,44,45,46,47,48,49,50,51,52].

## 5. Conclusions

The following research study concludes that geometric morphometric tools are a useful technique for the identification of small morphological variations in *T. infestans* head and wings, as well as highlighting population differentiation across the inter-Andean valleys and Chaco in Southern Bolivia, associated with geographical and environmental factors.

The *T. infestans* population of Huacaya/Imbochi, which is located in the dry Chaco, differ morphologically from other populations particularly by their plasticity to their geographical and environmental characteristics, as it is an area of dry plain with high temperatures greater than 30 °C and low humidity of less than 12%. Future analyses of pesticide concentration and environmental conditions could be useful in order to identify more factors that modulate morphological adaptation amongst populations [7].

## Figures and Tables

**Table 1:** Climatic information from the Military Institute of Geography for the four localities where *T. infestans* populations were sampled.

Locations	Annual Temperature	Annual Relative Humidity	Annual Pluvial Precipitation	Elevation.m.a.s.l.	Lat. Long.	Female	Male	Total
Yamparáez/ Sotomayor	18.7 °C	25%	448.2 mm	2990	Lat. 19°19′ S Long. 65°60′ O	12	16	28
Icla/Sumala	24.6 °C	45%	571.6 mm	2475	Lat. 19°26′ S Long. 64°50′ O	15	19	34
Monteagudo/ Cañón Largo	28.0 °C	30%	1017.0 mm	1130	Lat. 19°48′ S Long. 63°57′ O	11	11	22
Huacaya/Imbochi	28.2 °C	15%	489.1 mm	780	Lat. 20°37′ S Long. 63°10′ O	15	15	30
					Total	53	61	114

**Table 2:** Mahalanobis distance and Procrustes distance analysis for population in *Triatoma infestans*. HI: Huacaya/Imbochi, IS: Icla/Sumala, MC: Monteagudo/Cañón Largo, YS: Yamparáez/Sotomayor.

		Mahalanobis Distance			Procustres Distance		
		HI	IS	MC	HI	IS	MC
Head dorsal view	**IS**	2.1210 *			0.0162 *		
Head lateral view		3.0256 *			0.033 *		
wing		1.6149 *			0.0192 *		
Head dorsal view	**MC**	2.8458 *	2.5348 *		0.0196 *	0.0267 *	
Head lateral view		2.4708 *	1.5524 **		0.0327 **	0.0155 **	
wing		2.0329 *	2.1852 *		0.0199 *	0.0239 *	
Head dorsal view	**YS**	2.5317 *	2.0493 *	1.0784 ***	0.0177 *	0.0226 *	0.0084 ***
Head lateral view		2.4805 *	1.3624 **	1.3376 ***	0.0292 **	0.021 **	0.0183 ***
wing		1.5666 *	1.0957 **	1.9524 *	0.0193 *	0.0093 ***	0.0236 *

* *p* < 0.0001; ** *p* < 0.01; *** *p* > 0.0.
